# Tubal adenocarcinoma in a case report

**DOI:** 10.1016/j.ijscr.2022.107558

**Published:** 2022-08-27

**Authors:** N. Youssouf, Z. Sami, F. Watik, S. Sabir, Houssine Boufettal, Sakher Mahdaoui, Naima Samouh

**Affiliations:** Gynecology Department, Univesity Hospital Ibn Rochd, Faculty of Medicine and Pharmacy, Hassan II University of Casablanca, Morocco

**Keywords:** Trump, Cancer, Diagnosis, Treatment

## Abstract

**Introduction:**

Tumors of the uterine tube are rare pathologies representing less than 1 % of all gynecologic cancers; they are dominated by adenocarcinomas. Secondary metastatic forms are the most frequent, whereas primary tumors are very rare and represent only 10 %, which suggests that the fallopian tube is an organ with low oncogenic potential.

**Case report:**

We report the case of a patient followed in the gynecology department C of the CHU IBN ROCHD CASA for a primary tubal adenocarcinoma, with a history of breast cancer.

**Discussion:**

The diagnosis of its origin is difficult preoperatively, the treatment and staging are the same as for ovarian cancer.

**Conclusion:**

The treatment is also identical to the management of ovarian cancer, but their prognosis is better because they are most often diagnosed at an earlier stage.

## Highlights

• Primary tubal cancer is rare.
• The treatment is also identical to the management of ovarian cancer.
• MRI brings a great diagnostic interest.
• The prognosis depends on the FIGO stage.

## 1 Introduction

Primary tubal cancer is rare, often affecting postmenopausal women of unknown etiology, but it often occurs in the context of infertility, impoverishment, chronic tubal infection or on a genetic background (BRCA1/BRCA2 mutation) [1]. It was first described in 1847 by Renaud [2]. Its frequency does not exceed 1 %, the clinical signs are often dissociated, the preoperative diagnosis is difficult, with a prognosis that depends on the stage of the disease.

We report the case of a patient with a history of breast cancer and followed for primary adenocarcinoma of the tube that recurred 5 years after the initial totalization, through this case and a review of the literature we will try to support the risk factors and symptomatology as well as the management of these tumors. All our work has been reported in line with the SCARE criteria and guidelines [14].

## 2 Observation

This is a 62-year-old female patient, IGIP, postmenopausal, never had oral contraception, no notion of recurrent genital infection, She had undergone subtotal hysterectomy with bilateral adnexectomy in 2015 for a 10 cm right latero-uterine mass. The anatomopathological study showed a right intra tubal serous adenocarcinoma, grade III, without ovarian involvement or invasion of the epiplon. The patient received 6 courses of chemotherapy, the CA 125 was 192, a trachelectomy was performed 1 year later with non-specific fibrous remodeling with chronic exocervicitis, without signs of malignancy. 4 years later the patient consulted for chronic pelvic pain for 6 months, of increasing intensity, without sign of urinary or digestive compression. The clinical examination revealed a patient in good general condition, with good vitals, a supple abdomen, no clinically palpable mass, the vaginal slice was clean without lesions, the senological examination and the rest of the somatic examination were without particularity.

**Figs. 1 and 2:**
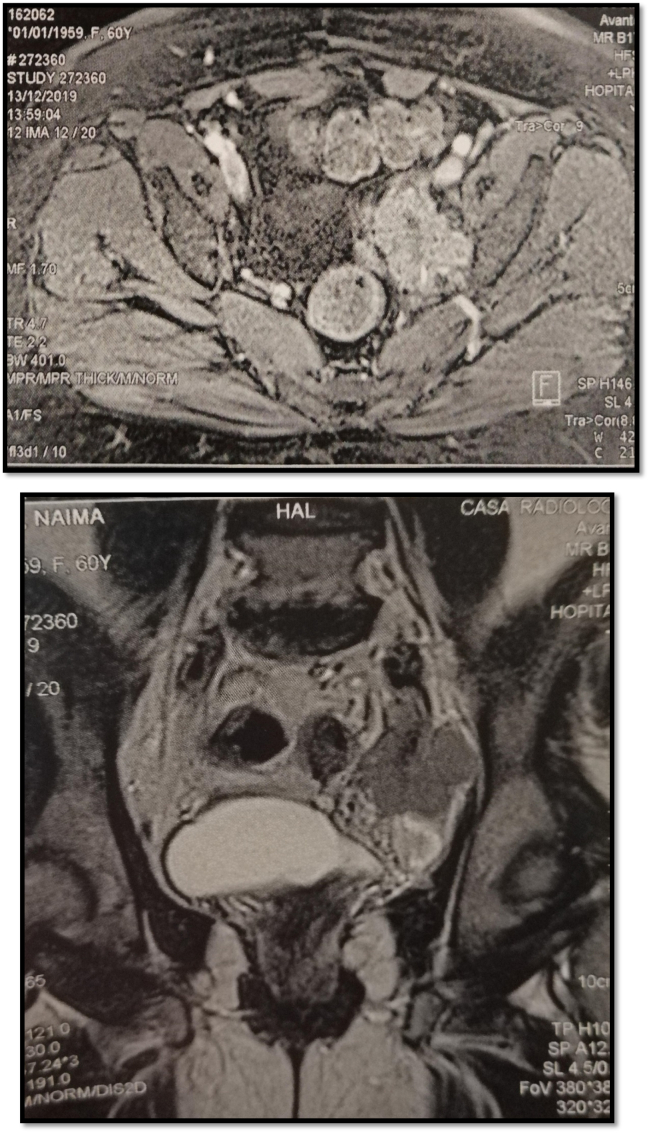
MRI appearance of recurrent tubal adenocarcinoma.

A pelvic MRI was performed showing a solid cystic mass measuring 52 mm in width, 42 mm in anteroposterior diameter, 64 mm in height, in contact with the bowel and the bladder without any sign of infiltration and without associated adenopathy (Figs. 1 and 2), CA 125: 321.6.

The exploration revealed a mass adherent to the right iliac vein without invading it, an excision was performed with simple postoperative follow-up with anatomopathological examination: tubal adenocarcinoma grade III, then the patient was referred to adjuvant chemotherapy.

## 3 Discussion

Tubal cancer is the rarest cancer of the genital tract representing 0.1 to 1.98 % of all gynecologic cancers [1]. It is a pathology that affects patients in the 5th decade with extreme ages of 18 and 87 years. Our patient is 62 years old and primiparous with a history of invasive breast carcinoma. Several authors have noted the association of tubal adenocarcinoma with impoverishment, infertility and chronic or genetic salpingitis, prompting a search for a deleterious BRCA1/2 chromosomal mutation, in our patient this association could not be sought due to lack of means [1].

The symptoms are not specific, often associating a pelvic mass, bleeding, and pain, but the pathognomonic triad is hydropstubaeprofluens associating abundant fluid loss, pelvic colic, and a mass is described in only 10 to 15 % of cases [4].

The first paraclinical examination to be requested is ultrasonography, which often shows the same aspect of epithelial carcinomas of the ovary in the form of a mixed adnexal mass, solid and cystic, hypervascularized on Doppler [5], [6]. Pelvic MRI is used for staging; the typical appearance of a tubal carcinoma is manifested by a hyperintense T2-weighted signal and a hypointense T1-weighted signal with a solid appearance. The tumor markers, mainly Ca125, are sensitive but not specific and are often elevated [7], [8].

Staging is based on ovarian cancer [4].

The treatment is also identical to the management of ovarian cancer, but their prognosis is better because they are most often diagnosed at an earlier stage [9]. The reference surgery is total hysterectomy, bilateral adnexectomy, omentectomy, pelvic and lomboaortic curage. Chemotherapy combining cisplatin and paclitaxel has the same indications as for ovarian cancer. Radiotherapy is abandoned because of its low efficacy [10], [11].

The prognosis depends on tumor size [4], stage [12], invasion [13], and the existence of a macroscopic postoperative residue [4]. The prognostic role of gene alteration of p53, Kras, c-erb-2 and immunolabeling of the tumor with CA125 is being evaluated [4].

The 5-year overall survival of tubal malignancies is estimated to be 44 % according to major series [9], [10], [11].

## 4 Conclusion

Tubal adenocarcinoma is a rare entity, of unknown etiology, underestimatedor often confused with ovarian pathology with which it shares the same treatment and staging, the positive diagnosis is difficult because the clinical picture is polymorphic, MRI brings a great diagnostic interest, the prognosis depends on the FIGO stage.

## Provenance and peer review

Not commissioned, externally peer-reviewed.

## Funding

None.

## Ethical approval

I declare on my honor that the ethical approval has been exempted by my establishment.

## Consent

Written informed consent for publication of their clinical details and/or clinical images was obtained from the patient.

## Author contribution

NABILA YOUSSOUF: Corresponding author writing the paper

SAMI ZINEB: writing the paper

WATIK.FEDOUA: writing the paper

SABIR SOUKAINA: writing the paper

BOUFETTAL Houssin: correction of the paper

MAHDAOUI Sakher: correction of the paper

SAMOUH NAIMA: correction of the paper

## Registration of research studies

researchregistry2464.

## Guarantor

DR YOUSSOUF NABILA.

## Declaration of competing interest

The authors declare having no conflicts of interest for this article.
